# Unplanned Emergency Department Visits Within 90 Days of Breast Reduction Surgery: A Retrospective Cohort Study

**DOI:** 10.1177/22925503251411906

**Published:** 2026-01-13

**Authors:** Olivia C. MacIntyre, Tamara D. Selman, Jo-Anne Douglas, Evan Nemeth, Julia M Harrison, Margaret E Wheelock, Emily M. Krauss

**Affiliations:** 1 Dalhousie Medical School, 12361 Dalhousie University Faculty of Medicine, Halifax, Canada; 2 Division of Plastic and Reconstructive Surgery, Department of Surgery, 3688 Dalhousie University, Halifax, Canada; 3 Department of Surgery, 12361 Dalhousie University, Halifax, Canada; 4 Performance and Analytics, Quality and System Performance, 432234 Nova Scotia Health, Halifax, Canada; 5 Division of Plastic Surgery, Department of Surgery, The IWK Health Centre, Halifax, Canada

**Keywords:** resource management, healthcare utilization, emergency department, reduction mammoplasty, quality improvement, amélioration de la qualité, département d’urgence, gestion des ressources, réduction mammaire, utilisation des soins de santé

## Abstract

**Introduction:** Emergency departments (EDs) have been under substantial strain in recent years, far exceeding current capacities and attracting media attention and calls for improved staffing and resources. A portion of patients presenting to EDs include postoperative patients. We sought to identify the proportion of patient presenting postoperatively to the ED after common plastic surgery elective procedures to identify target populations and presentations for diversion from the ED. This study investigates the incidence and etiology of postoperative ED presentations in the bilateral reduction mammoplasty (BBR) population, the most common general anesthetic plastic surgery procedure in our jurisdiction. **Methods:** This retrospective healthcare-utilization study collected all ED visits across a single province from April 2016 to March 2022 within 90 days postoperatively from BBR. Visit timing, frequency, chief complaint, diagnosis, and discharge destination from ED were recorded and analyzed for relationships to the index surgery and for predictable patterns. **Results:** Of the 452 patients who underwent bilateral breast reduction mammoplasty between 2016 and 2022, 75 (16.6%) patients presented to the ED within 90-day postoperatively. Of these 75 patients, 49 were directly related to the primary surgery (10.8% surgical ED return rate) for complaints of surgical site infection (36.7%), pain (30.6%), wound check (22.4%), swelling (20.4%), and bleeding (20.4%). **Conclusion:** After routine reduction mammoplasty, 10.8% of patients presented to the ED for surgery-specific concerns. This study describes targets to assist in diverting routine postoperative visits from the ED back toward the surgeon's office. Enhanced patient education, improved follow-up infrastructure, and pain control education are possible areas for intervention.

## Résumé

**Introduction :** Les urgences ont été soumises à de fortes pressions ces dernières années, qui dépassent largement les capacités actuelles, attirent l’attention des médias et nécessitent une amélioration de la dotation en personnel et des ressources. Une partie des patients qui consultent à l’urgence ont subi une opération récente. Les chercheurs souhaitaient établir quelle proportion de patients consultait à l’urgence après des interventions courantes et non urgentes en chirurgie plastique afin de déterminer les populations cibles à détourner de l’urgence. La présente étude a porté sur l’incidence et l’étiologie des consultations postopératoires à l’urgence parmi la population des personnes qui ont subi une réduction mammaire bilatérale (RMB), l’intervention de chirurgie plastique en anesthésie générale la plus courante de la région sociosanitaire. **Méthodologie :** La présente étude rétrospective d’utilisation du système de santé a permis de recueillir toutes les consultations à l’urgence dans une seule province entre avril 2016 et mars 2022 dans les 90 jours suivant une RMB. Les chercheurs ont analysé le moment et la fréquence des consultations, la principale doléance, le diagnostic et la destination du congé de l’urgence pour établir les relations avec l’opération de référence et les profils prévisibles. **Résultats :** Des 452 patientes qui ont subi une réduction mammaire bilatérale entre 2016 et 2022, 75 (16,6%) ont consulté à l’urgence dans les 90 jours suivant l’opération. Chez ces 75 patientes, 49 des consultations étaient directement reliées à l’opération primaire (10,8% de taux de retour à l’urgence à cause de l’opération) à cause d’une infection au foyer de l’opération (36,7%), de douleur (30,6%), d’une vérification de la plaie (22,4%), d’un œdème (20,4%) ou de saignements (20,4%). **Conclusion :** Après une réduction mammaire courante, 10,8% des patientes consultent à l’urgence en raison de préoccupations liées à l’opération. Cette étude décrit les cibles pour contribuer à réorienter les consultations postopératoires à l’urgence vers le cabinet de chirurgie. Une meilleure éducation des patients, une meilleure infrastructure de suivi et de l’éducation sur le contrôle de la douleur font partie des secteurs possibles d’intervention.

## Introduction

Canadian emergency departments (EDs) have seen increasing demands for patient care, with average wait times to see a physician often spanning multiple hours.^1^ As of 2021, provincial healthcare expenditures have continued to rise in their allotment to EDs in comparison to previous years.^2^ This increase in healthcare cost correlates to usage, with individuals presenting for care exceeding current capacities, compounded by limited access to primary care physicians.^3^ A substantial number of ED visits are patients who recently underwent surgery for postoperative concerns or complications.^4^ Given the overwhelming demand seen within EDs nationwide, nonemergent postoperative complications represent a potential target for diversion.

Breast surgeries, including bilateral breast reduction mammoplasty (BBR), are some of the most common surgeries worldwide, ranking in the top 10 most performed procedures by plastic surgeons.^5^ BBR is the most common breast surgery procedure performed in our province by plastic surgeons, now primarily as an outpatient procedure.^6^ In outpatient surgery, there is a higher need for comprehensive postoperative instructions for emergency complications and access to postoperative assessment for both serious and benign postoperative concerns. The purpose of this study was to evaluate healthcare utilization of the ED in BBR patients within 90 days of surgery, to identify areas of intervention to better support postoperative plastic surgery patients and reduce ED burden.

## Methods

### Ethical Approval

This project was a Quality Improvement Project exemption from Nova Scotia Health and IWK Health Centre Research Ethics Board and given permission to publish.

### Study Population

Patients who underwent BBR at 2 hospitals within a single provincial care zone (Nova Scotia Health (NSH) Central Zone) between April 1, 2016, and March 31, 2022, with a subsequent presentation to any ED across the province within 90 days of the primary surgery were included. These 2 hospitals are the primary centers offering BBR for the province during the study period. For patients who had multiple BBR surgeries between April 2016 and March 2022, only the primary surgery was included.

### ED Visit Data Collection

ED visits were identified from the NSH ED Provincial ED Information System datasets, System for Tracking and Analysis of Emergency Room, and Meditech to provide ED descriptive variables (ED site, ED length of stay [LOS], days from surgical discharge, broad description of reason for ED visit based on triage) and filtered by primary procedure as BBR using the NSH administrative datasets, Discharge Abstract Database (DAD) and the National Ambulatory Care Reporting System (NACRS). Patients who underwent a BBR were classified using the primary procedure Canadian Classification of Health Intervention (CCI) code of 1.YM.78.LA-XX-E “repair by decreasing breast size using a simple excisional technique with local flap (eg, inferior, vertical, or central).”^7^

**Figure 1.**
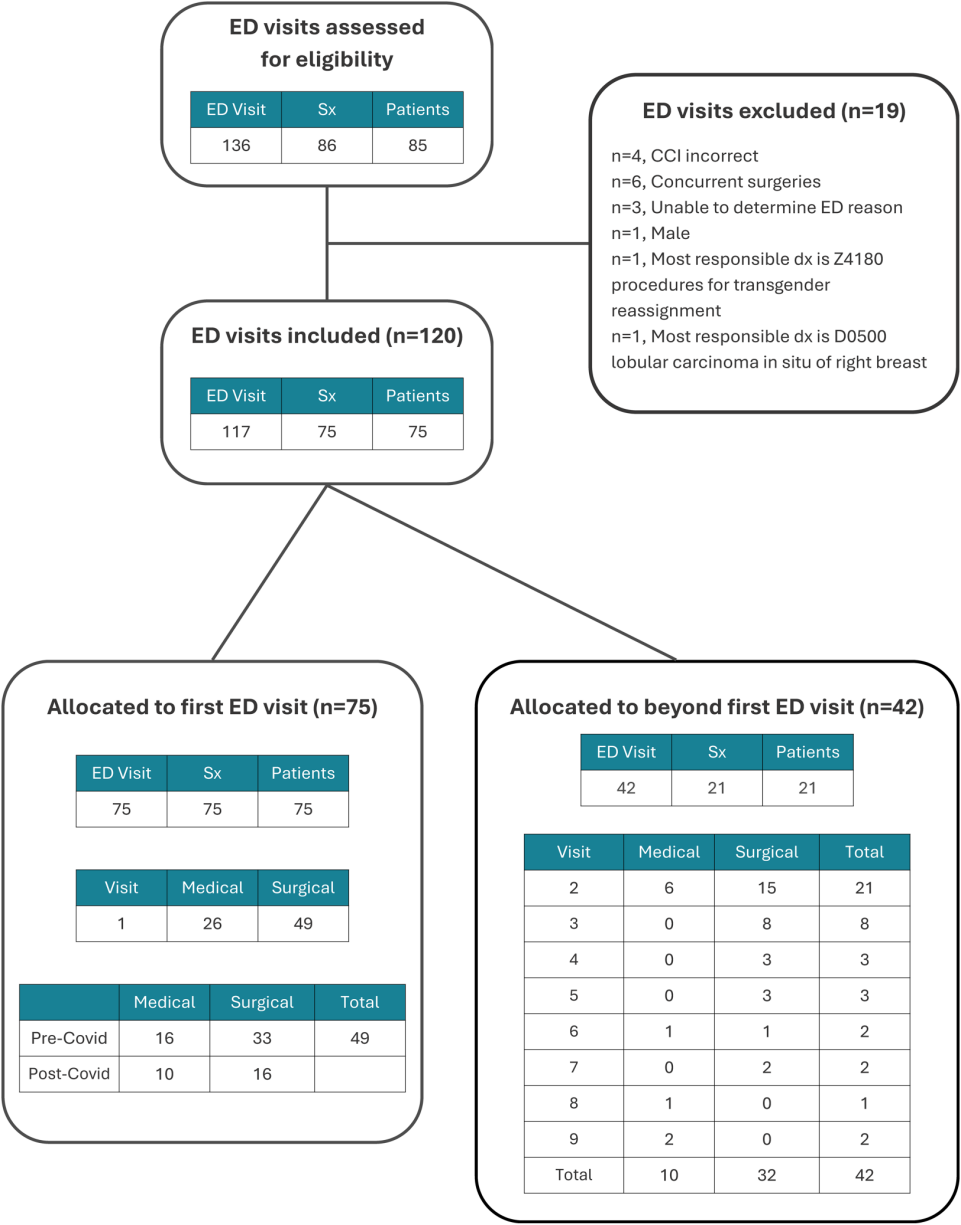
Consort Diagram of Postoperative Emergency Department Visit Selection.

Given that a single patient may have multiple ED visits within 90 days postoperatively, only the number of unique patients that had a primary BBR and their first ED visit within 90 days were analyzed (Figure 1). Visits were excluded from the study if the chart reviewer noted an incorrect CCI code, if the administrative data had no documentation of the ED visit, if the reason for ED visit could not be determined, if the procedure was for male gynecomastia or for gender affirming surgery, if the underlying diagnosis was breast cancer, or if a concurrent surgery was performed perioperatively (such as abdominoplasty). If a patient had a revision surgery within the study timeframe, only the first surgery was included as the index surgery and only ED visits prior to this revision surgery were included. If a second or greater ED visit occurred for the same patient within 90-days postoperatively, the first ED visit was analyzed for timing, chief complaint, diagnosis, and discharge destination. Second and subsequent visits are not included in the main analysis but were evaluated separately.

Only individuals with provincial healthcare numbers are included in the databases. DAD and NACRS datasets were cross-referenced for patient and surgical demographic descriptive variables (age, sex, patient residence urban/rural, hospital, surgeon, day of stay, LOS). Urban/rural status was determined from postal codes.^8^ Charts were reviewed by 5 independent investigators to review the reason for ED visit, clinical diagnosis, discharge destination, and to characterize the visit as either a primary medical or surgical cause. Anonymous data was recorded in a secure REDCap^®^ database (version 14.1.6, Vanderbilt University, Nashville, TN, USA) for analysis.

### Statistical Analysis

Descriptive statistics including frequencies, proportions, means, ranges and standard deviation were calculated using SAS software (Version 9.4 of the SAS System for Windows. Copyright ^©^ 2016 SAS Institute Inc. [Cary, NC, USA]).

## Results

Between 2016 and 2022, 458 bilateral breast reduction mammoplasties were performed on 452 patients in 2 centrally located hospitals which provide the majority of plastic surgery care in the province. Of these patients, 16.6% (*n* = 75) presented to an ED within 90 days of discharge (Figure 1). Of the 75 patients presenting to an ED, the median age was 42.7 (±15.4) and the majority resided in an urban area (50, 77.3%) (Table 1). Most patients had a primary care provider at the time of ED visit according to ED records (72, 96.0%). Of the 75 patients presenting to the ED, 59 had an outpatient BBR, while 16 were admitted postoperatively and discharged on the first postoperative day after the index procedure. Outpatient BBR versus overnight admission reflects surgeon preference and changing practice patterns over the data acquisition period. As an incidence of BBR patients returning to the ED, 22.5% of inpatient BBR patients (16/71) and 15.15% of outpatient BBR patients (59/381) presented to the ED within 90 days after surgery.

**Table 1.** Demographics of Breast Reduction (BBR) Patients From 2016 to 2022, and Those Presenting to the ED (ED Rebound) Within 90 Days of Surgery.

Characteristics of BBR patients and procedures 2016-2022	Overall
BBR procedures (*n*)	458
BBR patients (*n*)	452
Inpatient BBR patients (*n*)	71
Outpatient BBR patients (*n*)	381
ED rebounds within 90 days:	
Patients (*n*)	75
Age (y), mean (SD)	42.7 (15.4)
Gender, *n* (%)	
Female	75 (100)
Male	0
Patient geography, *n* (%)	
Rural	17 (22.7)
Urban	58 (77.3)
Primary care status, *n* (%)	
Has primary care	72 (96.0)
No primary care	3 (4.0)
Sx stay type, *n* (% incidence ED rebound)	
Inpatient	16/71 (22.5%)
Outpatient	59/381 (15.5%)
Total ED visits, *n*	
Medical causes	26
Surgical causes (related to BBR)	49
COVID timeline *n*/total patients (% incidence ED rebound)	
Pre-COVID (before March 2022)	33/270 (12.2%)
Post-COVID (after March 2022)	16/184 (8.7%)
Discharge to ED visit (postoperative days)	
Surgical causes (related to BBR)	
Mean (*SD*)	17.0 (14.5)
Median (IQR)	15.0 (5.0-26.0)
Length of ED stay per patient (hours)	
Surgical causes (related to BBR)	3.0 (2.9)
Mean (SD)	
Post-op education *n* (%)	
Eligible (preventable ED visit)	19 (25.3%)
Ineligible	26 (34.7%)
Indeterminate	30 (40.0%)

*Note.* ED, emergency department.

**Figure 2.**
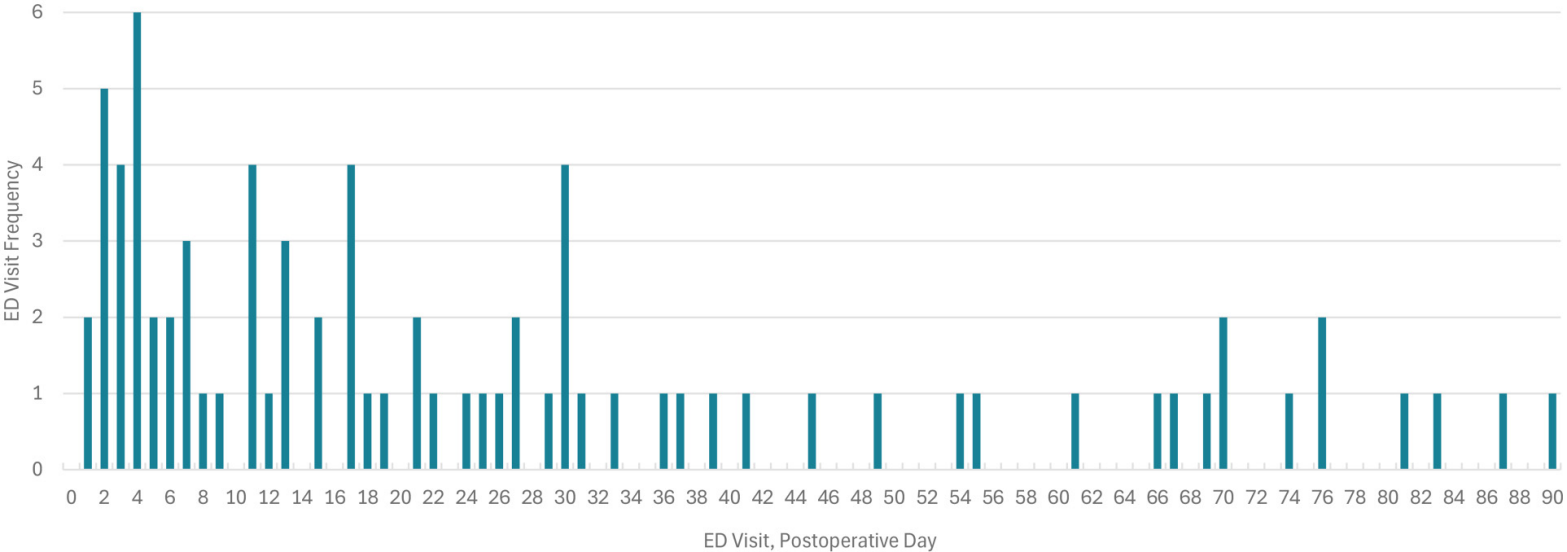
Emergency Department Visit Frequency by Day Following Breast Reduction Mammoplasty.

**Figure 3.**
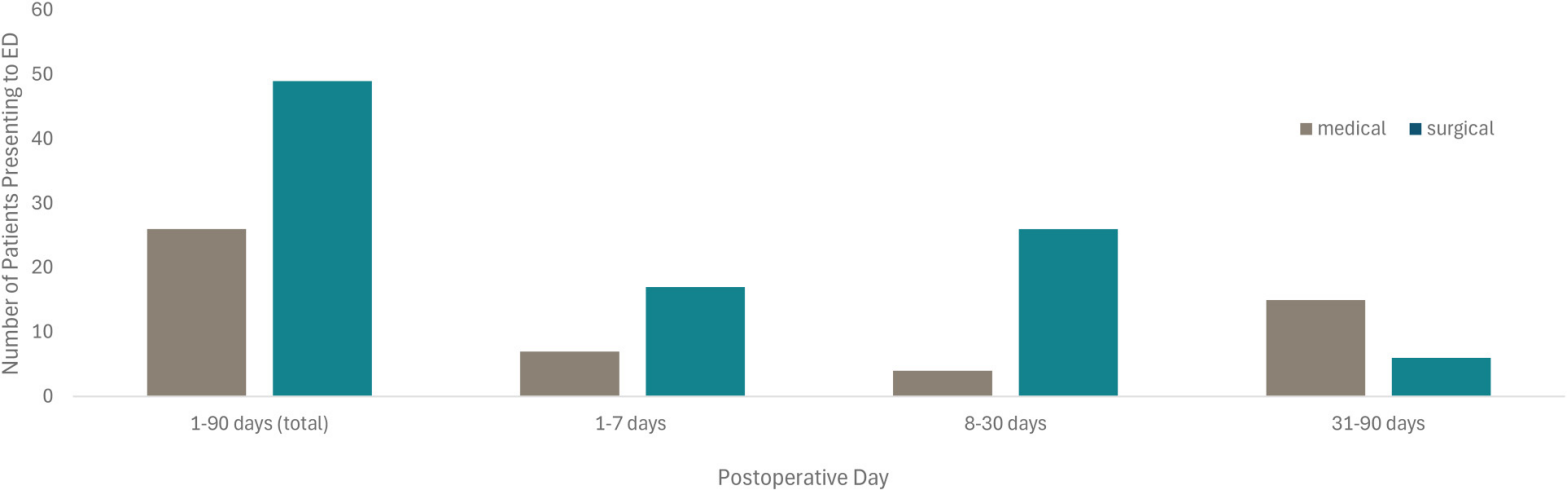
Total Number of Patients Presenting to the Emergency Department for Medical or Surgical Reasons Following Bilateral Reduction Mammoplasty Within 90 Days.

The median day for patients to present to an ED for surgical-related reasons was postoperative day 17 (Table 1, Figure 2). The single day with the highest frequency of ED visits was postoperative day 4. Following postoperative day 40, ED visits became less frequent overall. For ED visits from 1 to 7 days and 8 to 30 days following discharge, presentations were overwhelmingly related to the primary surgery (70.8% and 86.7%, respectively) (Table 2) (Figure 3). Visits between postoperative days 31 and 90 were more likely to be for medical causes unrelated to the primary surgery (71.4%). All ED presentations due to medical causes were reviewed and determined to be unrelated to the BBR surgery. The visits to the ED for medical reasons (*n* = 26) were subsequently excluded from further analysis.

**Table 2.** Details of Postoperative Breast Reduction ED Visit Types.

ED visit details	1-90 days (overall)	1-7 days	8-30 days	31-90 days
Total ED visits, *n* (%)				
Medical	26 (34.7)	7 (29.2)	4 (13.3)	15 (71.4)
Surgical	49 (65.3)	17 (70.8)	26 (86.7)	6 (28.6)

*Note.* ED, emergency department.

Of the 75 first ED visits, 49 (65.3%) of these were coded as surgical and were directly related to BBR, representing an overall surgical ED return rate of 10.8% for BBR. On average, patients presenting for surgical reasons spent 3.0 h in the ED from triage to either discharge (*n* = 43, 87.8%) or admission (*n* = 3, 6.1%; Table 1). Nearly 89% of patients presenting to the ED after BBR were discharged home (discharged home *n* = 60) (80.0%), discharged home with follow-up *n* = 7 (9.3%). A total of 5 patients (6.67%) were admitted to hospital from the ED for hematoma or infection. All admitted patients were hemodynamically stable according to the ED record.

**Figure 4.**
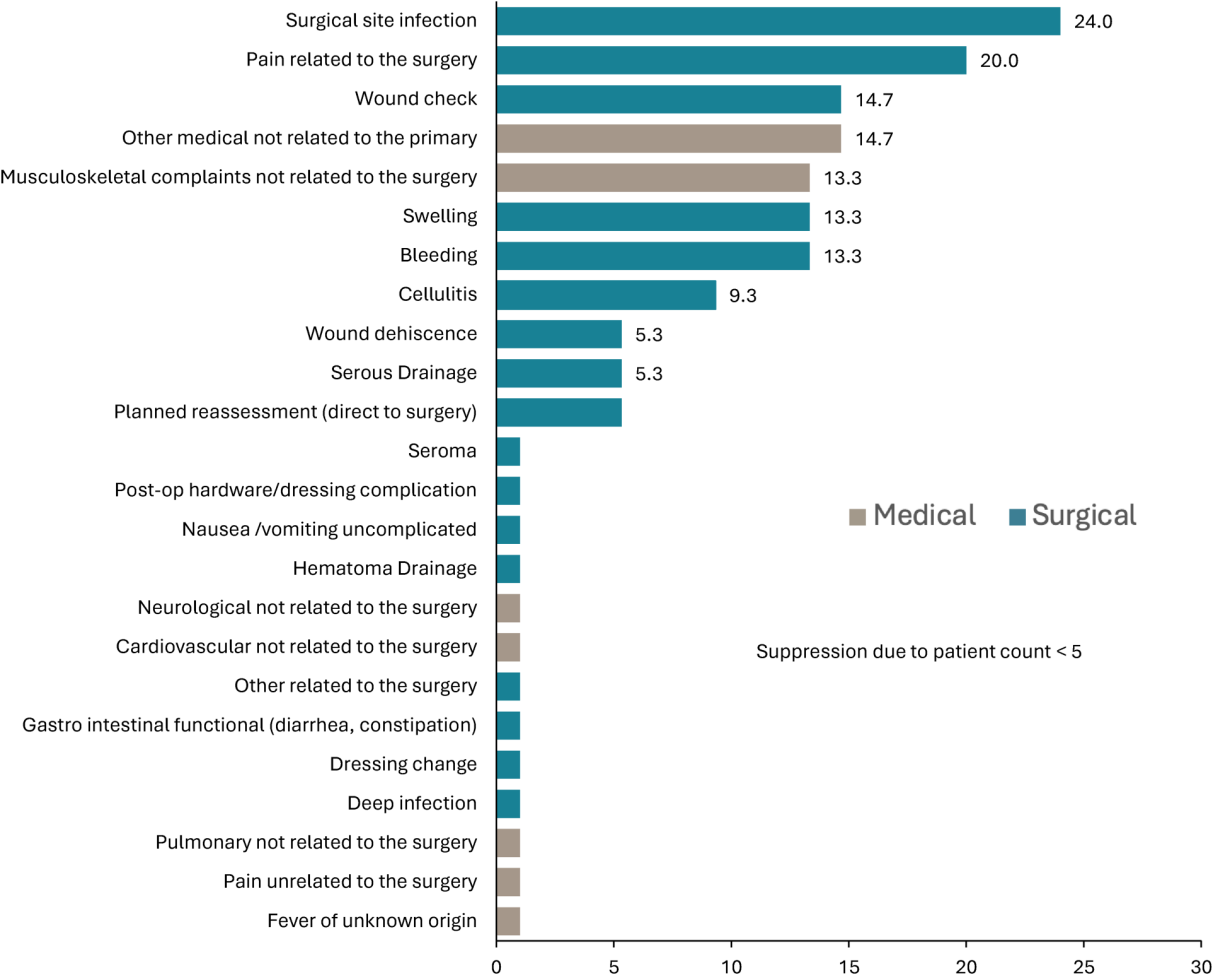
Reason for Post–Breast Reduction Presentation to the Emergency Department by Diagnosis as a Percentage of Total Reasons for Presentation.

Surgical site infection (*n* = 18, 4.0% incidence, 36.7% of surgical visits) was the most common diagnosis for ED presentation related to BBR (as defined as prescription of antibiotics; Figure 4). Pain related to the surgery (*n* = 15, 3.3% incidence, 30.6% of surgical ED visits), wound checks (*n* = 11, 2.4% incidence, 22.4% of surgical ED visits), swelling (*n* = 10, 2.2% incidence, 20.4% of surgical ED visits), and bleeding (*n* = 10, 2.2% incidence, 20.4% of surgical ED visits) were other common reasons for ED visits related to BBR. Only 2 patients required a surgical intervention following their visit to the ED, both to drain a hematoma (*n* = 2, incidence 0.4% incidence). Less common reasons included cellulitis (*n* = 7), serous draining or wound dehiscence (*n* = 8), and dressing complications (*n* = 2). Enhanced postoperative education was identified as a possible intervention in 19 surgery-related ED visits (38.7% of those presenting to the ED for surgical causes, representing 4.2% of the BBR total population) (Table 1).

## Discussion

In this comprehensive review of ED utilization following one of the most common operations in plastic surgery, 10.8% of BBR patients presented to an ED for reasons related to their primary surgery within 90 days postoperatively. Based on a detailed review of ED records, nearly 4% of the BBR population presented to the ED for reasons best dealt with by their primary surgeon, or mitigated through patient education resources. This data represents province-wide ED utilization after BBR performed at 1 of 2 hospitals that provide the majority of provincial breast surgical care. The rate of ED rebound reported here is high compared to published literature. Published ED rebound rates for breast reduction mammoplasty are scarce, with one study out of the United States reporting 5% to 13%.^9^ Similar ED return rates in outpatient mastectomy and cancer-related lumpectomy are reported as 5% to 7% within the first week to 30 days postoperatively.^10,11^ Our data capture a larger postoperative window up to 90 days within a publicly funded healthcare system which differs from the U.S. data which tend to be restricted to 30 days and capture individual hospital or insurance networks.

The most common reason for BBR patients to present to the ED was surgical site infection, representing 38.7% of those returning to the ED, suggesting a 4.0% population incidence of surgical site infection in the total BBR population (Figure 4). While our study defined surgical site infection as the prescription of antibiotics rather than positive bacterial culture, this rate is well within or below national averages.^12,13^ However, this value cannot be used to report the overall postoperative infection rates in BBR as it does not capture patients presenting to clinic. Further, the hospital database charting sets the definition of “infection” to the prescription of antibiotics which may overestimate the true postoperative infection rate to include those who were prescribed antibiotics out of caution, or those where delayed T-junction wound healing is misinterpreted as infection. A further 3.3% of BBR patients presented to the ED for uncomplicated pain and 2.4% for wound checks, all of which could have been managed outside the ED (such as the attending surgeon's office). Patients reporting postoperative pain are within reported metrics; 22% of BBR patients are reported to experience pain beyond 3 months postoperatively.^14^ While surgical site infection and postoperative bleeding may be reasonable to present to the ED, only 3 patients out of the 75 presenting to the ED required admission to hospital (for operative hematoma drainage or IV antibiotics) and all were stable. This indicates that majority of the patients in this study population could have been seen by their primary surgeon outside of the ED if better educated on what presentations should prompt an ED visit and what can be managed through the surgeon's office.

Previous studies evaluating patient decision-making to present to the ED have identified convenience (eg, hours of operation, familiarity, location) and perceived severity of the concern.^15,16^ However, most breast reduction patients (77.3%) resided in urban areas, geographically close to their attending surgeon's office as well as primary care resources. Primary care status did not influence ED visits, as over 96% of BBR patients had a primary care provider at the time of ED presentation. The high ED utilization may reflect perceived urgency of the concern or the availability of the surgeon or primary care provider. Given our retrospective study design, we were unable to determine how many of these patients may have sought care outside the ED but were unable to contact their surgeon or family doctor. A study in 2010 from one local primary care clinic reported that mean wait time from appointment request to visit was 13.7 days.^17^ It is possible that our metric for primary care status is an overestimate; however, our health authority's patient records are updated by the patient and registration personnel upon registration to be seen in the ED.

Of the surgical-related ED visits, 19 (38.8%) were identified as visits that could have been mitigated through enhanced postoperative education or greater postoperative patient resources. These visits included reasons such as uncomplicated pain, uncomplicated bleeding, wound checks, dressing changes, and uncomplicated swelling. Enhanced knowledge sharing of normal postoperative changes (swelling and bleeding) and the stages of wound progression must be prioritized to support patients until they can be assessed by their primary surgeon and successfully diminish the number of patients presenting to the ED. This notion is supported by Pelt et al (2018), who determined that patient education not only reduced patient's perception of pain but also decreased the number of readmissions and reoperations.^18^ Another study found that patients receiving less postoperative education than anticipated were more likely to report complications in comparison to patients receiving their expected level of education.^19^ As per standard of care, our department provides preoperative and postoperative verbal and written communication by the surgical team at consultation and peri-operatively and in postoperative recovery by a nurse. However, our results highlight that an enhanced and updated patient educational strategy should be employed at our center. T-junction wound dehiscence can be confused for wound infection and is a common cause of patient anxiety. Improved visual resources, provided to patients at appropriate time points, may enhance the postoperative course and decrease the need to seek additional care.

Postoperative concerns are best managed by the attending surgeon and in many health authorities there is a formalized expectation of surgeon availability (or equivalent representative) to assess and treat all postoperative patients presenting with concerns related to surgery. Comprehensive conversations to set patient expectations for postoperative recovery while clearly defining when patients should present to the ED are warranted in our population. Our center continues to provide standard postoperative care for all patients. This includes postoperative instructions for incision and pain management as well as ensuring office availability and prompt follow-up appointments. Despite this, patients still present to the ED following breast reduction for wound checks and pain management. Providing patients with reliable surgeon's office contact information and assurance that short-notice support including in-person appointments, phone calls, and video calls will be available may help alleviate the tendency to present to the ED. Currently patients do not have access to a surgeon on call over the phone after hours, as this service is not provided across the province. Having a phone number for a surgeon on call may divert patients away from the ED, but the primary driver of ED visits in the population is still unclear.

The most common day for ED return for breast reduction patients was postoperative day 4, with an overall average presentation at day 17 despite having a standard follow-up appointment booked with their surgeon around 2 weeks (Table 1). One option to limit early return to ED may be implementing earlier postoperative phone calls to ease patient concerns in advance of the standard comprehensive 2-week follow-up. Understanding a patient's individualized postoperative needs such as their ability to change dressings and administer analgesic medication independently may also provide insight into the required level of education and support. Beyond standard postoperative instructions, providing holistic information for postoperative care such as ensuring adequate time off work to recover, balanced nutrition to optimize healing, and education on gentle exercises and stretches to improve pain and mindfulness to enhance surgical recovery.^20^

**Figure 5.**
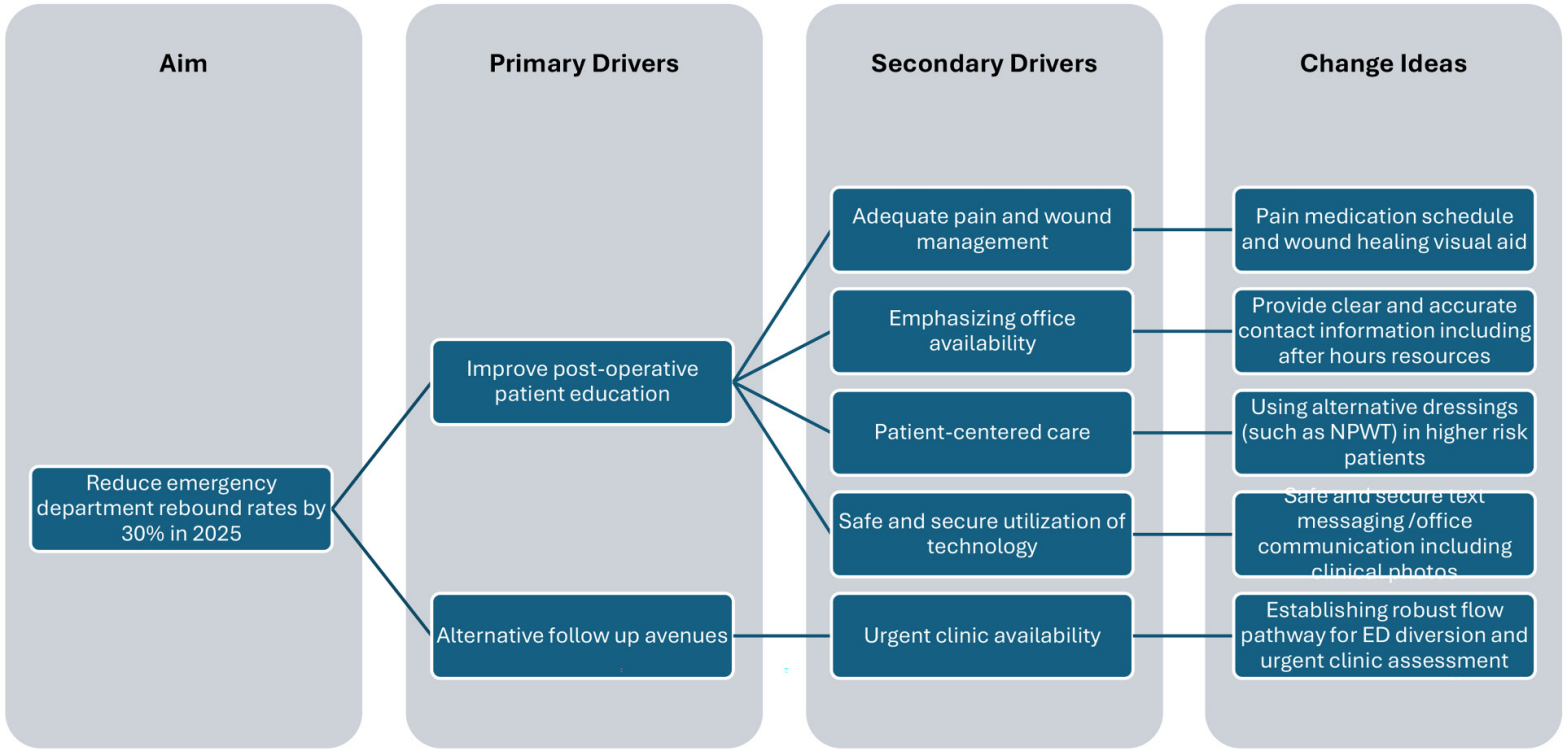
Driver Diagram of Current Initiatives to Reduce Postoperative Unplanned Emergency Department Visits.

There are several factors we are seeking to incorporate to better address postoperative needs (Figure 5). Emphasizing surgeon office availability during daytime hours to address patient concerns is one tool that may reduce ED burden. Technological tools that provide virtual assistance and real-time communication with surgeon's offices are other possible solutions to better address patient postoperative concerns, particularly with rural-residing patients or those with accessibility concerns. However, these have mixed results on utility, reduction of in-person visits, and patient satisfaction in plastic surgery patients.^21^ Other options include group patient education seminars where the surgical team provides standard preoperative information using audiovisual aids in a time-efficient manner. These classes have been shown to improve patient physical impairment, pain, knowledge, and preoperative anxiety.^22^

### Limitations

This study is a comprehensive analysis of regional ED utilization following breast reduction surgery for a broad geographic area within a single-payer public healthcare system but excludes patients who received procedures at a private clinic or in another zone. This study does not include the factors that lead patients to choose the ED rather than their surgeon, primary care provider, or alternative, and whether there were gaps in patient understanding of postoperative recovery. Additionally, factors of interest that were not detailed in this analysis are if patients were more likely to present to EDs in the evening or on weekends as well as when the patient's last point of contact had been with their surgeon prior to ED presentation. Future prospective studies could focus on patient-perception factors that lead to ED presentation following breast surgery, as well as if enhanced surgeon availability may mitigate these ED visits. If a patient presented to the ED more than once within the 90-day period, their later visits were excluded from the primary analysis. This was to avoid counting the same concern more than once in the dataset, thus the total number of ED visits was higher than the number of patients presenting to the ED, as we report in this study.

This is a retrospective health utilization study that depends on the accurate coding of primary procedure and cross-referencing multiple databases to obtain patient, surgical, and presentation demographics. The quality of the data and its temporal relationship is dependent on coding and classification. Clinical records were reviewed as scanned PDFs of paper charts, which limited our ability to easily search data points and procedure classifications within the electronic medical record systems. Clinical records of ED visits frequently had limited information regarding examination and patient perspectives, limiting the inferences we could make regarding the urgency and severity of patient concerns and their perspectives in seeking care. Additionally, as only ED visits tied to the primary procedure were reviewed, this study does not provide insight into the true procedure-specific complication rate in this population, as patients not presenting to the ED were not included in the analysis but may have visited their surgeon with a complication.

## Conclusion

Postoperative BBR patients returned to the ED within 90 days at a rate of 10.8% for causes specific to surgery. Patients presented for concerns of surgical site infection, pain related to surgery, and wound checks. These visits occurred on average 17 days following surgery, and usually between 1 and 30 days. All presentations were reasonable to be seen in the surgeon's office instead of the ED. These results represent an opportunity in postoperative patient healthcare utilization through patient education and resources to divert patients away from the ED for timely assessment by their surgical team for nonemergency postoperative concerns.
